# A causal variant rs3769823 in 2q33.1 involved in apoptosis pathway leading to a decreased risk of non-small cell lung cancer

**DOI:** 10.20892/j.issn.2095-3941.2022.0068

**Published:** 2022-09-02

**Authors:** Xu Zhang, Na Qin, Jingyi Fan, Chang Zhang, Qi Sun, Yayun Gu, Meng Zhu, Erbao Zhang, Juncheng Dai, Guangfu Jin, Hongxia Ma, Zhibin Hu, Hongbing Shen

**Affiliations:** 1 Department of Epidemiology, Center for Global Health, School of Public Health, Nanjing Medical University, Nanjing 211166, China; 2 Jiangsu Key Lab of Cancer Biomarkers, Prevention and Treatment, Collaborative Innovation Center for Cancer Personalized Medicine, Nanjing Medical University, Nanjing 211166, China; 3 Health Management Center, The Affiliated Suzhou Hospital of Nanjing Medical University, Suzhou Municipal Hospital, Gusu School, Nanjing Medical University, Suzhou 215008, China; 4 Research Units of Cohort Study on Cardiovascular Diseases and Cancers, Chinese Academy of Medical Sciences, Beijing 100730, China

**Keywords:** Non-small cell lung cancer, chromosome 2q33.1, risk, caspase-8, apoptosis

## Abstract

**Objective:**

Although our previous genome-wide association study (GWAS) has identified chromosome 2q33.1 as a susceptibility locus for non-small cell lung cancer (NSCLC), the causal variants remain unclear. The aims of this study were to identify the causal variants in 2q33.1 and to explore their biological functions in NSCLC.

**Methods:**

CCK-8, colony formation, EdU incorporation, Transwell, and quantitative real-time polymerase chain reaction assays were applied to examine variant function. The tumor xenograft model was used to examine variant function *in vivo*. Caspase-8 activity assays, flow cytometry analysis, and co-immunoprecipitation assays were used to explore the molecular mechanism.

**Results:**

The missense variant rs3769823 (A > G), which caused the substitution of lysine with arginine at amino acid 14 in caspase-8 (caspase-8K14R), was identified as a potential causal candidate in 2q33.1. Compared with the wild type caspase-8 (caspase-8WT) group, the caspase-8K14R group had higher expression of caspase-8 and cleaved caspase-8. Caspase-8K14R inhibited the proliferation and metastasis of human lung cancer cell lines *in vitro*. Moreover, caspase-8K14R repressed lung cancer cell growth *in vivo*. Mechanistically, caspase-8K14R was more sensitive than caspase-8WT to tumor necrosis factor-related apoptosis-inducing ligand (TRAIL)-mediated apoptosis and showed higher binding of caspase-8 and FADD.

**Conclusions:**

These results suggested that rs3769823 is the causal variant in chromosome 2q33.1 and is involved in an apoptosis pathway, leading to a decreased risk of NSCLC.

## Introduction

Lung cancer is one of the most commonly diagnosed malignant tumors with the highest mortality rate^1–3^. Non-small cell lung cancer (NSCLC) is the major histological type of lung cancer, accounting for approximately 85% of all lung cancer cases^4,5^. Increasing evidence suggests that environmental factors, such as tobacco consumption, air pollution, and occupational exposures, contribute to the risk of lung cancer^6^. In addition, genetic factors play an important role in lung cancer susceptibility^7–9^.

In recent decades, genome-wide association studies (GWASs) have reported 45 lung cancer susceptibility loci^9,10^. Our recent work has identified 6 additional loci for NSCLC^8^. However, most of the identified genetic variants are tagged single nucleotide polymorphisms (SNPs), and the underlying causal variants are unknown^7^. Therefore, identifying causal variants from these loci and elucidating their potential biological functions remain a major challenge but may help unlock the potential of GWASs^11,12^.

Functional annotations are critical in identifying putative causal variants in complex diseases^13,14^. To identify the potential functional variants in NSCLC susceptibility loci and to elucidate the underlying susceptibility mechanisms, we have recently integrated large-scale genotype, transcriptomic, and epigenomic datasets, and have identified 803 functional target genes and associated credible risk variants for NSCLC. Of all defined target genes, caspase-8, located in 2q33.1, had the strongest functional evidence, and the missense variant rs3769823 (c.41A > G, p.Lys14Arg) was considered the potential causal variant at 2q33.1^15^. However, the biological function and mechanism of rs3769823 in NSCLC remain unclear. Therefore, this study was aimed at verifying the potential biological role of rs3769823 in the tumorigenesis of NSCLC *via* biological experiments.

## Materials and methods

### Cell lines and cell cultures

The human lung cancer cell lines (A549 and SPCA1) were obtained from the Institute of Biochemistry and Cell Biology of the Chinese Academy of Sciences (Shanghai, China). Cell lines were cultured in RPMI 1640 basic medium (GIBCO-BRL, Invitrogen, Carlsbad, CA) or DMEM (GIBCO-BRL, Invitrogen) supplemented with 10% fetal bovine serum (FBS, Gibco, USA) and 1% antibiotics (100 U/mL penicillin and 100 μg/mL streptomycin) (Invitrogen). These cell lines were incubated at 37 °C in a humidified atmosphere of 5% CO_2_.

### Plasmid construction

The full-length DNA fragments of the transcript variant G (i.e., isoform 7 in this study)^15^ of caspase-8 containing rs3769823[A] or rs3769823[G] were cloned into the pcDNA3.1(+) vector. The wild type caspase-8 (caspase-8WT) group was treated with a plasmid containing rs3769823[A], and the caspase-8K14R group was treated with a plasmid containing rs3769823[G]. Sequences of all constructed plasmids were verified before experiments.

### Transfection

Cells were seeded in a 6-well plate for 24 h. The constructed plasmid was transiently transfected into A549 and SPCA1 cells with X-tremeGENE™ HP DNA Transfection Reagent (Roche) according to the manufacturer’s protocol.

### Western blot assays

At 48 h post-transfection, cells were lysed with RIPA lysis buffer (Keygen BioTECH) with protease inhibitor cocktail (MedChemExpress). Cell protein lysates were separated with 4%–20% SurePAGE and then transferred to 0.2 μm PVDF membranes (Millipore, MA, USA). Membranes were blocked in 5% BSA albumin fraction V (Biofroxx) and incubated with specific antibodies (anti-caspase-8: 1:1000, A19549, ABclonal; anti-cleaved-caspase-8: 1:1000, 9496S, Cell Signaling Technology; and anti-β-actin: 1:10000, 66009-1-Ig, Proteintech). Protein bands were visualized with a molecular imager (Bio-Rad, Hercules, CA).

### Cell proliferation assays

Cell viability was measured with a CCK-8 Cell Counting Kit (Vazyme, China) according to the manufacturer’s protocol, and 5-ethynyl-2′-deoxyuridine (EdU) incorporation assays were performed with a Cell-Light™ EdU Apollo 567 In Vitro Kit (Ribobio, Guangzhou, China) according to the manufacturer’s protocol. The cells were observed under a confocal laser scanning microscope (ECLIPSE-Ti, Nikon, Japan). The EdU incorporation rate was used to assess proliferation [EdU-positive cells (red cells)/total DAPI-positive cells (blue cells)], and the cells were counted in Image-Pro Plus (IPP) 6.0 software (Media Cybernetics).

### Colony formation assays

Cells were seeded in 6-well plates and incubated at 37 °C. After visible colonies appeared, cell colonies were washed with saline, fixed with methanol, and stained with crystal violet (Beyotime). The number of clones was counted in imaging software.

### Cell migration assays

Cell migration assays were performed with Costar Transwell plates (6.5 mm diameter insert, 8.0 mm pore size, polycarbonate membrane, Corning Sparks, MD). The upper chambers were filled with cells in 300 μL of RPMI 1640 or DMEM supplemented with 1% FBS. The lower chambers were filled with 600 μL of RPMI 1640 or DMEM supplemented with 10% FBS. Then the cells were incubated at 37 °C in an atmosphere with 5% CO_2_ for 24 h. Afterward, the cells were fixed with methanol and stained with crystal violet. Subsequently, the membranes were washed and dried. Five random fields in the membranes were captured with an optical microscope (ECLIPSE TS100, Nikon), and the cells were counted in imaging software. All experiments were repeated 3 times independently.

### RNA extraction and quantitative real-time polymerase chain reaction (qRT-PCR)

Total RNA was extracted from the harvested cells with TRIzol reagent (Invitrogen). RNA was reverse transcribed to obtain complementary DNA with HiScript II Q RT SuperMix for qPCR (Vazyme, China). The qRT-PCR analyses were performed with TB Green Premix Ex Taq (Takara, China). The mRNA expression results were normalized to the expression of β-actin. The primers are presented in **Supplementary Table S1**.

### Lentiviral production and infection

For lentiviral production (caspase-8WT and caspase-8K14R), the full-length complementary DNA of isoform 7 of caspase-8 containing rs3769823[A] or rs3769823 [G] was cloned into the pSLenti-SFH-EGFR-P2A-Puro-CMV-3xFLAG-WPRE vector. The lentivirus was purchased from OBIO Technology (Shanghai, China). For infection, cells were stably transfected with the lentivirus. Afterward, the cells were cultured in medium containing puromycin dihydrochloride (MedChemExpress). Finally, monoclonal stable transgenic cell lines were selected for *in vivo* assays.

### Mouse xenograft tumor model

BALB/c nude mice were maintained in specific-pathogen-free conditions. Cells expressing caspase-8WT and caspase-8K14R were injected subcutaneously into the left and right armpits, respectively. The tumor volumes were measured as length × width^2^ × 0.5. At the indicated number of days after injection, the mice were killed, and the tumor weights were measured and analyzed. Animal care and handling procedures were performed in accordance with the National Institutes of Health’s Guide for the Care and Use of Laboratory Animals and were approved by the Committee on the Ethics of Animal Experiments of Nanjing Medical University (Nanjing, China) (approval number: IACUC-1812039-2).

### Caspase-8 activity assays

The cells were homogenized in lysis buffer and centrifuged at 16000 × g for 15 min at 4 °C. The supernatants were collected to detect the activity of caspase-8 with a caspase-8 activity assay kit (C1152, Beyotime) according to the manufacturer’s instructions. The activity of caspase-8 was evaluated according to the absorbance measured at 405 nm. Then the results were normalized to the protein concentrations and are shown as percentages of the specific value.

### IC50 assays

The IC50 values of cell lines for tumor necrosis factor (TNF)-related apoptosis-inducing ligand (TRAIL) were determined with CCK8 assays. Cells were seeded in 6-well plates. At 24 h post-transfection, the cells were seeded in 96-well plates. Afterward, the cells were treated with the indicated concentrations of TRAIL for 72 h, then subjected to CCK8 assays.

### Flow cytometry analysis

Transfected cells were harvested after 48 h of transfection and treated with different concentrations of TRAIL. The cells were stained with Annexin V-FITC and PI Staining Solution with an Annexin V-FITC/PI Apoptosis Detection Kit (Vazyme, China) according to the manufacturer’s protocol. Then the cells were analyzed with a flow cytometer (FACScan, BD Biosciences) equipped with Cell Quest software (BD Biosciences). Differences between the caspase-8WT and caspase-8K14R groups treated with different concentrations of TRAIL were compared.

### Co-immunoprecipitation (co-IP) assays

Co-IP assays were performed with a Pierce Classic Magnetic IP/Co-IP Kit (Pierce, Thermo) according to the manufacturer’s protocol. Pretreated cells were rinsed twice with precooled PBS and lysed with ice-cold IP lysis/wash buffer on ice for 15 min, then subjected to centrifugation. Supernatants were incubated with anti-FADD (14906-1-AP, Proteintech) or control IgG at room temperature, followed by prewashed protein A/G magnetic beads at 4 °C overnight on a rotator. After extensive washing with IP lysis/wash buffer, the supernatant was collected, and 100 μL of protein loading buffer was added. The mixture was heated at 100 °C for 10 min, and the beads were separated and used for Western blot analysis (anti-FADD: 1:200, 14906-1-AP, Proteintech; anti-caspase-8: 1:100, 9746S, Cell Signaling Technology).

### Statistical analysis

All data were assayed by comparison of the mean ± SD with Student’s *t* test for different groups. All the tests were two-sided, and *P* values < 0.05 were considered statistically significant. General analyses were performed in GraphPad Prism 6 software.

## Results

### Caspase-8K14R has higher expression of caspase-8 and inhibits cell proliferation and metastasis in NSCLC

To explore the role of rs3769823 in NSCLC, A549 and SPCA1 cell lines were tested by Sanger sequencing. SNP rs3769823 was a homozygous mutation, and the genotype in A549 and SPCA1 cell lines was [GG] (**Supplementary Figure S1**). We chose these 2 cell lines for follow-up experiments.

**Figure 1:**
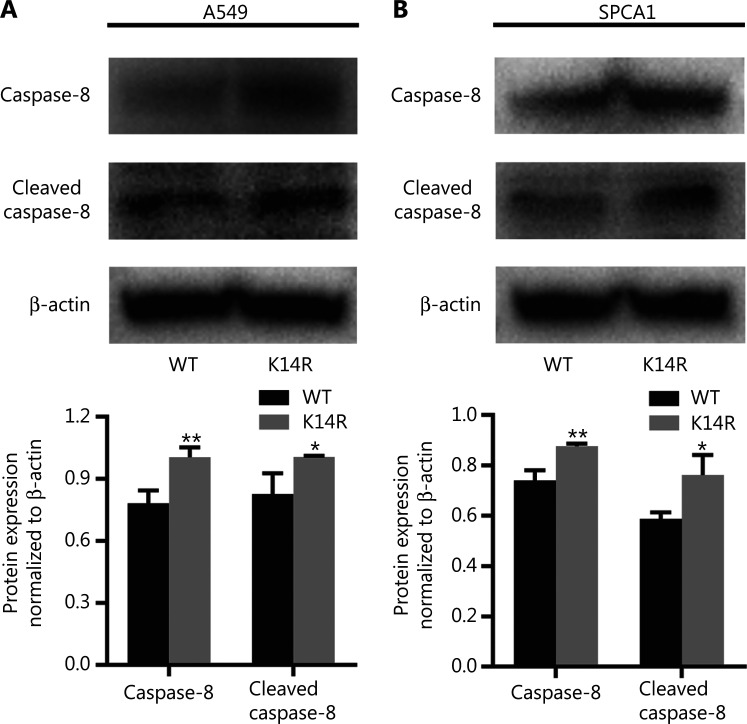
Caspase-8K14R has higher expression than caspase-8WT. (A, B) The expression of caspase-8 (55 kDa) and cleaved caspase-8 (18 kDa) was detected by Western blot after transfection with plasmids containing the rs3769823[A] or rs3769823[G] allele into A549 and SPCA1 cells (*P < 0.05, **P < 0.01).

**Figure 2:**
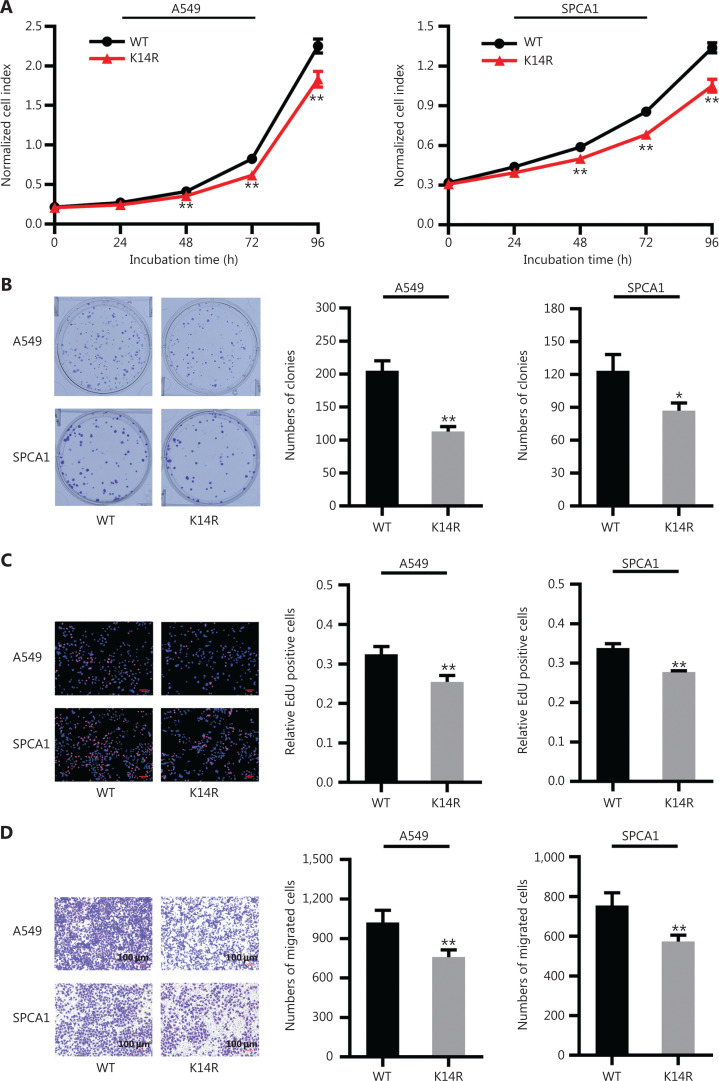
Caspase-8K14R inhibits lung cancer cell proliferation and metastasis. (A) CCK8 assays were performed to observe the proliferation of A549 and SPCA1 cells after transfection with plasmids containing the rs3769823[A] or rs3769823[G] allele. (B) Colony formation assays were used to investigate the differences in colony formation ability after transfection with the constructed plasmids into A549 and SPCA1 cells. (C) EdU incorporation assays were performed to assess the proliferation of A549 and SPCA1 cells after transfection with the constructed plasmids. (D) Transwell assays were conducted to determine the migration of A549 and SPCA1 cells (*P < 0.05, **P < 0.01).

Given that rs3769823 generates the substitution of lysine with arginine at amino acid 14 in caspase-8 (caspase-8K14R)^15^, we first examined the effect of the K14R alteration on caspase-8 expression and found that the expression levels of caspase-8 and cleaved caspase-8 were higher in the caspase-8K14R group than the caspase-8WT group (**Figure 1A and 1B**). Because caspase-8 initiates extrinsic apoptosis^16^, caspase-8K14R may be involved in an apoptosis-associated pathway. Therefore, we examined the effect of caspase-8K14R on cell phenotypes. CCK8 assays revealed that caspase-8K14R inhibited the lung cancer cell proliferation than caspase-8WT (**Figure 2A**). Similarly, colony formation assays showed that the cell colony-forming ability was lower in the caspase-8K14R group (**Figure 2B**). The EdU incorporation assays also confirmed the effect of caspase-8K14R on decreasing the cell proliferation rate (**Figure 2C**). Transwell assays indicated that the migration ability was repressed in the caspase-8K14R group (**Figure 2D**). These findings suggested that caspase-8K14R may play an important role in the tumorigenesis of NSCLC.

### Caspase-8K14R regulates the expression of genes associated with proliferation and apoptosis

**Figure 3:**
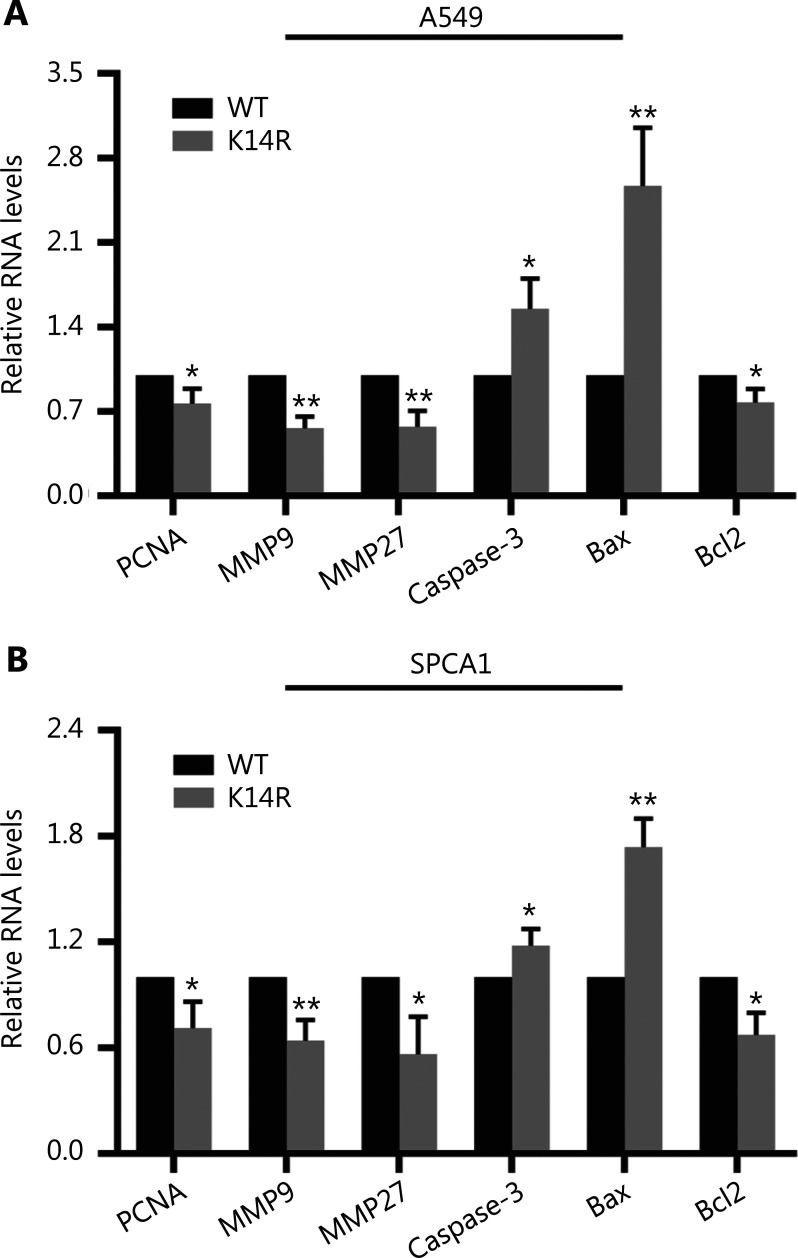
Caspase-8K14R regulates the expression of genes associated with proliferation and apoptosis. (A, B) qRT-PCR assays were performed to detect the mRNA levels of relevant genes in A549 and SPCA1 cells after transfection with the constructed plasmids (*P < 0.05, **P < 0.01).

Next, we detected the expression of 6 molecular marker genes in the caspase-8K14R group and caspase-8WT group, including proliferation- and metastasis- and apoptosis-associated genes. The caspase-8K14R group had lower mRNA levels of proliferating cell nuclear antigen (PCNA), matrix metalloproteinase 9 (MMP9), matrix metalloproteinase 27 (MMP27), and the anti-apoptosis factor Bcl2, and had higher mRNA levels of caspase-3 and Bax (**Figure 3A and 3B**). These results indicated that caspase-8K14R down-regulates the expression of genes associated with promoting proliferation and metastasis, and up-regulates the expression of genes associated with promoting apoptosis.

### Caspase-8K14R represses cell growth in vivo

**Figure 4:**
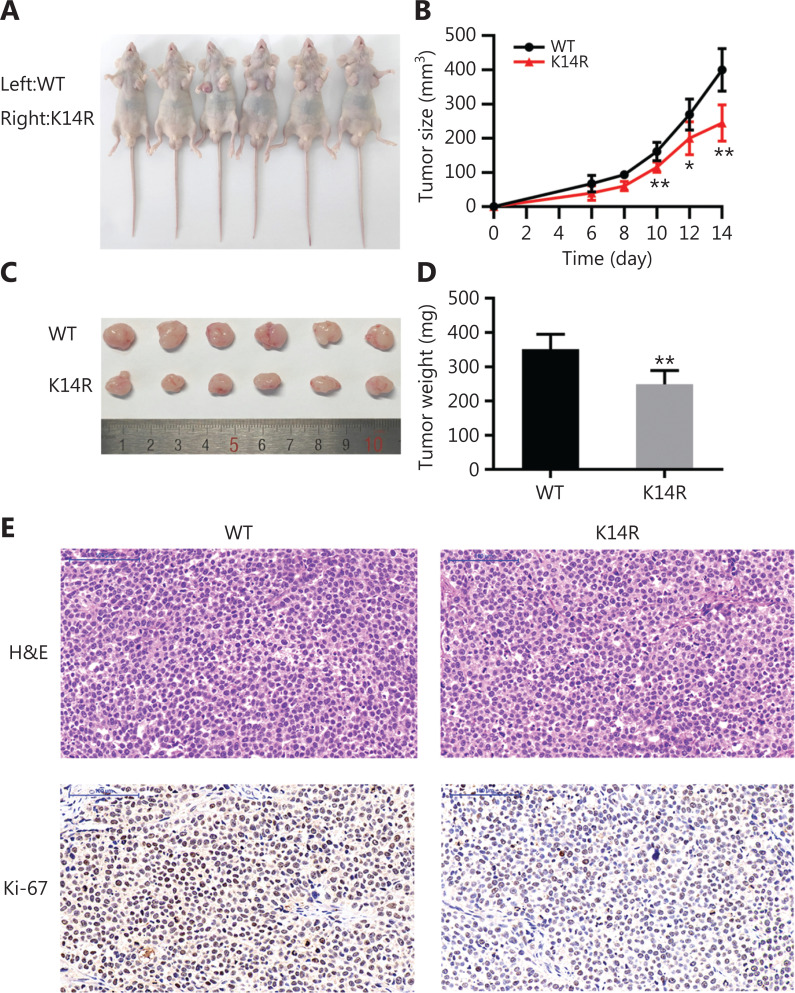
Caspase-8K14R represses cell growth *in vivo*. (A, B) Nude mice were injected with the SPCA1 cell line overexpressing the rs3769823[A] or rs3769823[G] allele. Images and growth curves of xenograft tumors are shown. (C, D) Tumor volumes and weights were calculated. (E) Tumor sections with H&E staining and IHC staining with antibodies against Ki-67 are shown (*P < 0.05, **P < 0.01).

To explore the role of caspase-8K14R *in vivo*, we stably transfected cells with lentivirus and selected monoclonal stable transgenic cell lines for *in vivo* assays. The cells were injected subcutaneously into the bilateral armpits of BALB/c nude mice. Less growth of xenograft tumors was observed in the caspase-8K14R group than the caspase-8WT group (**Figure 4A and 4B**). Moreover, the average tumor weight at the end of the experiment was lower in the caspase-8K14R group (**Figure 4C and 4D**). Caspase-8K14R had lower Ki-67 positivity (**Figure 4E**). Therefore, caspase-8K14R regulates tumor growth *in vivo*.

### Caspase-8K14R is more sensitive to TRAIL-mediated apoptosis

**Figure 5:**
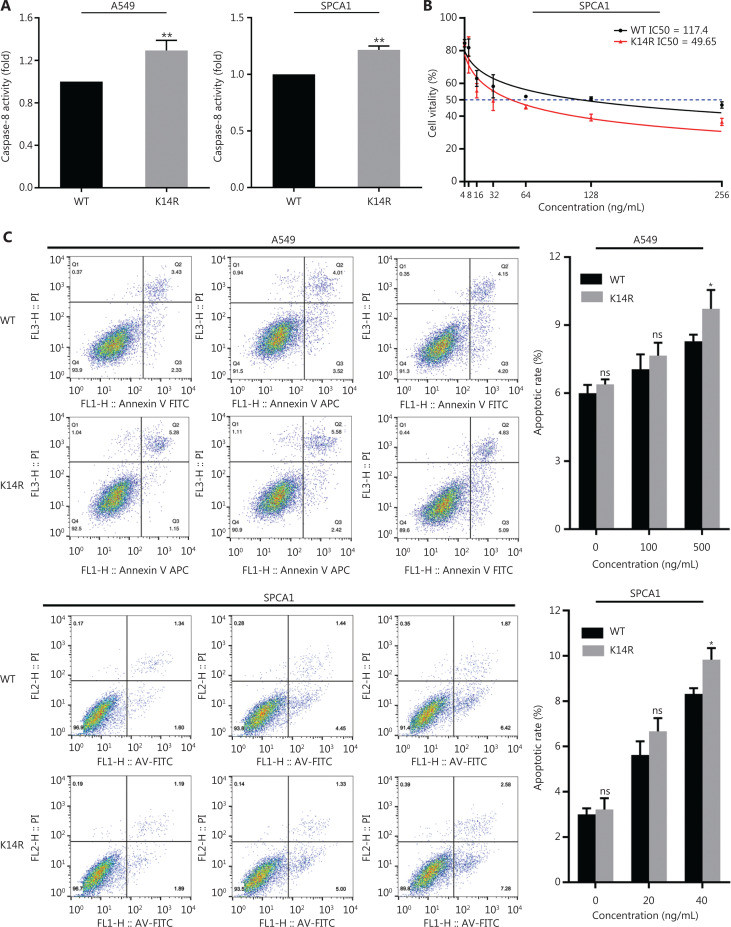

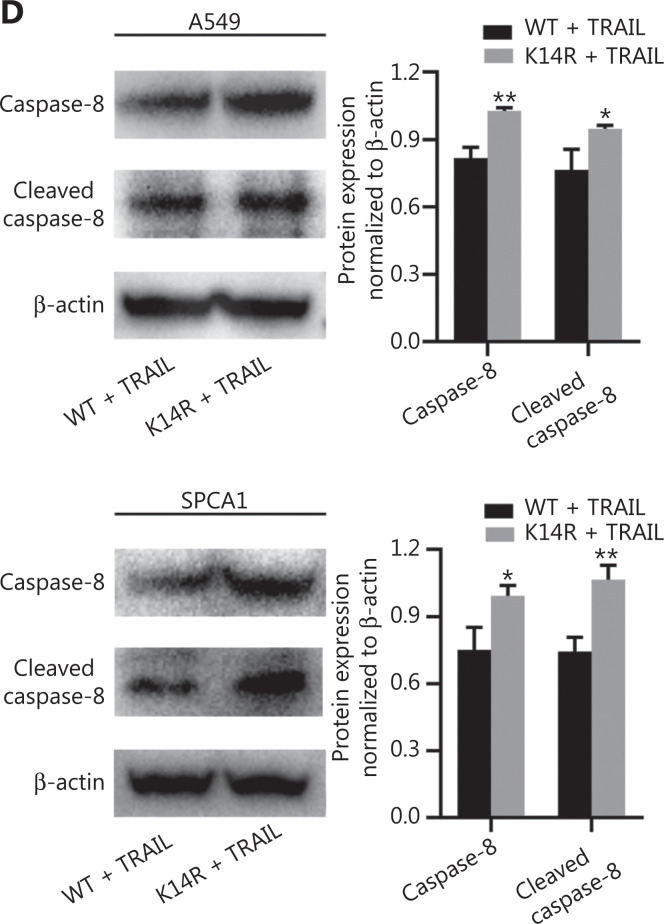
The roles of caspase-8K14R in TRAIL-mediated apoptosis. (A) Effects of the K14R alteration on caspase-8 activity in A549 and SPCA1 cell lines. (B) IC50 assays were preformed, and the IC50 values of the cell lines treated with TRAIL were determined with CCK8 assays in SPCA1 cells. (C) Flow cytometry was performed to detect the apoptosis rates of A549 and SPCA1 cells treated with different concentrations of TRAIL. (D) The expression of caspase-8 (55 kDa) and cleaved caspase-8 (18 kDa) was detected by Western blot after transfection with plasmids containing the rs3769823[A] or rs3769823[G] allele into A549 and SPCA1 cells treated with different concentrations of TRAIL (*P < 0.05, **P < 0.01).

To explore the effect of the K14R alteration on caspase-8 activity, we tested the activity of caspase-8 in both the caspase-8K14R and caspase-8WT groups. Caspase-8K14R showed higher caspase-8 activity than caspase-8WT (**Figure 5A**). An important role of caspase-8 is triggering the extrinsic apoptosis pathway when TRAIL binds TRAIL-R^17–19^. Thus, we sought to determine the effects of caspase-8K14R on apoptosis after treatment with TRAIL. The IC50 value in the caspase-8WT group was found to be 117.4, whereas that in the caspase-8K14R group was 49.65 in SPCA1 (**Figure 5B**). Caspase-8K14R was more sensitive than caspase-8WT to TRAIL-mediated apoptosis. In addition, we performed flow cytometry analysis to determine the different effects on apoptosis in both the caspase-8K14R and caspase-8WT groups. When treated with a suitable concentration of TRAIL, caspase-8K14R showed a higher apoptosis rate than caspase-8WT. Although the difference did not reach statistical significance, caspase-8K14R also showed a higher apoptosis rate than caspase-8WT after treatment with 0 ng/mL or lower concentrations of TRAIL (**Figure 5C**). We then examined the expression of caspase-8 in the caspase-8K14R and the caspase-8WT groups treated with a suitable concentration of TRAIL, and found that the expression of caspase-8 and cleaved caspase-8 was higher in the caspase-8K14R group than the caspase-8WT group (**Figure 5D**). Overall, the above results indicated that caspase-8K14R may inhibit the proliferation of lung cancer cells by affecting the apoptosis pathway.

### The K14R alteration results in greater binding of caspase-8 to FADD, thus affecting cell apoptosis in NSCLC

**Figure 6:**
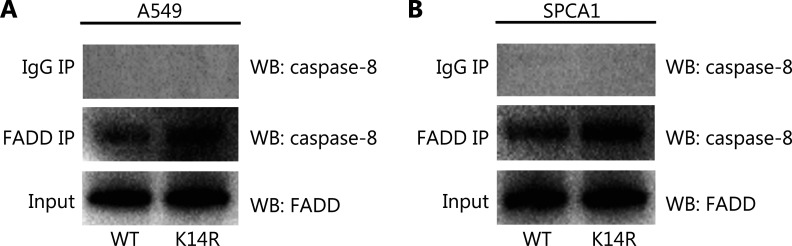
The K14R alteration increases binding of caspase-8 to FADD. (A, B) A549 and SPCA1 cell lines were transfected with rs3769823[A] or rs3769823[G]-expression plasmids. Then the cells were subjected to immunoprecipitation with anti-FADD antibody, followed by immunoblotting with anti-caspase-8.

Fas associated death domain (FADD) binds caspase-8 and forms the death inducing signaling complex (DISC), which plays a major role in the transmission of TRAIL-induced apoptosis signals^18,20,21^. To determine the role of caspase-8K14R in the TRAIL-induced apoptosis pathway, we tested differences in the binding of caspase-8 and FADD by performing co-immunoprecipitation assays. When cell lysates were subjected to immunoprecipitation with anti-FADD, followed by immunoblotting with anti-caspase-8, the binding of caspase-8 to FADD was higher in the caspase-8K14R group than the caspase-8WT group (**Figure 6A and 6B**), thus suggesting that caspase-8K14R affects the recruitment of caspase-8 to FADD.

## Discussion

**Figure 7:**
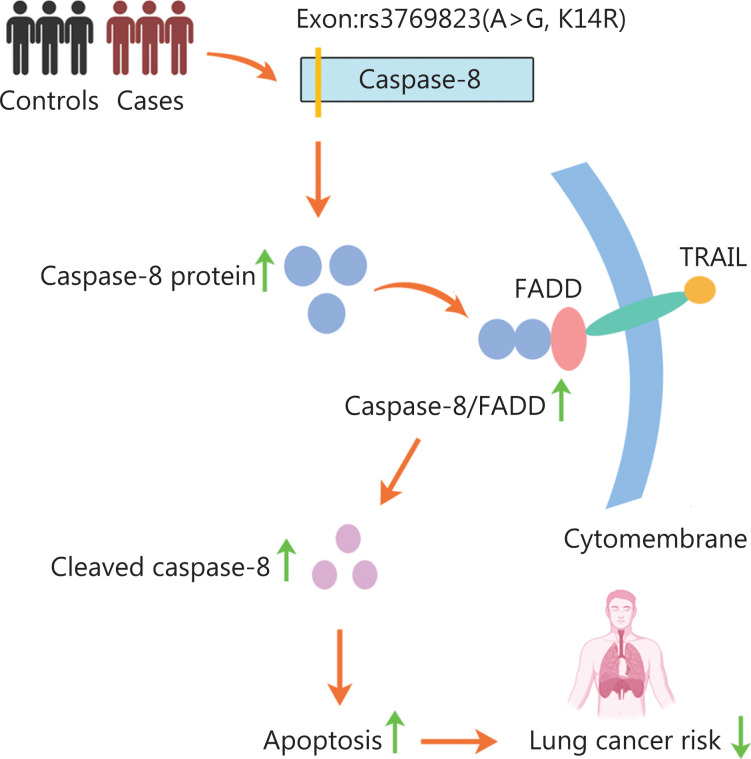
Schematic model of this study. The missense variant rs3769823 was the causal variant in 2q33.1. The K14R alteration resulted in higher expression of caspase-8, and greater binding of caspase-8 and FADD, thus promoting TRAIL-mediated apoptosis and inhibiting NSCLC tumorigenesis (image created with BioRender.com).

As a robust approach, GWASs have been used to identify a series of genomic loci associated with lung cancer risk; however, the causal variants and potential biological functions remained unclear. In the present study, we identified rs3769823, a missense variant, as the causal variant at 2q33.1. Caspase-8K14R, compared with caspase-8WT, showed higher expression of caspase-8 and binding of caspase-8 to FADD, thus subsequently promoting TRAIL-mediated apoptosis and inhibiting NSCLC tumorigenesis (**Figure 7**).

Some evidence has indicated a relationship between rs3769823 and cancers, including lung cancer, breast cancer, esophageal squamous cell carcinoma, and basal cell carcinoma^15,22–25^. Notably, Lin et al.^25^ have conducted a meta-analysis of GWAS and found that the rs3769823[A] allele increases the risk of basal cell carcinoma independently of the [G] allele. This finding is consistent with our results indicating that the rs3769823[G] allele is protective against lung cancer. In addition, to our knowledge, the present study is the first report showing the biological functions of rs3769823 in NSCLC.

Missense mutations can cause amino acid substitutions, which may lead to crucial structural alterations, affect protein stability, or perturb protein binding interfaces. These alterations may impair protein functions^26,27^. Stability, as a fundamental property of proteins, is strongly affected by missense mutations^28–31^. Singh et al.^32^ have revealed that the missense mutation Lys18Asn (K18N) in dystrophin might trigger X-linked dilated cardiomyopathy by decreasing protein stability, increasing protein unfolding, and perturbing protein structure. Additionally, Liao et al.^33^ have found that PERP-428 variants (rs648802, c.428C > G, p.Pro143Arg) affect lung cancer risk by differentially regulating p53 protein stability and PTEN/MDM2/p53-mediated antioxidant activity. In the present study, the caspase-8K14R group had higher expression of caspase-8 than the caspase-8WT group. We speculated that this higher expression might have been due to influences on protein stability and unfolding. The mechanism through which caspase-8K14R affects protein levels needs to be further studied.

Caspase-8 belongs to the cysteine aspartyl protease (caspase) family. Caspases drive apoptosis by cleaving aspartic acid peptide bonds, thus resulting in cell death^17^. Here, we found that caspase-8K14R inhibited malignancy and increased apoptosis rates. In addition, we detect the mRNA levels of relevant molecular markers in the caspase-8K14R and caspase-8WT groups. PCNA is an essential protein that participates in a variety of processes of DNA metabolism and is involved in cell survival^34^. Matrix metalloproteinases (MMPs) are zinc dependent proteolytic metalloenzymes. Inhibiting MMP9 has anticancer effects, and MMP27 is associated with invasion and migration^35,36^. Bax and caspase-3 are pro-apoptotic proteins, and Bcl2 suppresses apoptosis^37–39^. We verified that caspase-8K14R down-regulated the expression of genes associated with promoting proliferation and metastasis, and up-regulated the expression of genes associated with promoting apoptosis.

Caspase-8 is the key initiator caspase in death receptor-induced apoptosis induced by external stimuli, such as TRAIL^18,40,41^. TRAIL belongs to the tumor necrosis factor superfamily, and its binding to TRAIL receptors initiates apoptosis^42^. The adaptor proteins FADD and caspase-8 are then recruited, thus forming DISC^18,20,21^. Subsequently, caspase-8 is cleaved, forming active caspase-8 (heterotetramer - p18_2_ - p10_2_), which cleaves and activates caspase-3, which in turn executes the cell apoptosis pathway^18^. We found that caspase-8K14R, compared with caspase-8WT, inhibited NSCLC malignancy, was more sensitive to TRAIL-mediated apoptosis, and showed higher binding between caspase-8 and FADD. Hence, caspase-8K14R appears to suppress lung cancer malignancy by affecting the cell apoptosis pathway.

## Conclusions

In conclusion, we demonstrated that rs3769823 is the causal variant in chromosome 2q33.1 according to functional experimental verification. Our findings provide additional evidence supporting an in-depth understanding of the genetic susceptibility mechanisms in NSCLC.

## References

[1] Sung H, Ferlay J, Siegel RL, Laversanne M, Soerjomataram I, Jemal A (2021). Global Cancer Statistics 2020: GLOBOCAN estimates of incidence and mortality worldwide for 36 cancers in 185 countries. CA Cancer J Clin.

[2] Zhao M, Feng J, Tang L (2021). Competing endogenous RNAs in lung cancer. Cancer Biol Med.

[3] Chen X, Shen C, Wei Z, Zhang R, Wang Y, Jiang L (2021). Patient-derived non-small cell lung cancer xenograft mirrors complex tumor heterogeneity. Cancer Biol Med.

[4] Bender E (2014). Epidemiology: The dominant malignancy. Nature.

[5] Moreno-Rubio J, Ponce S, Alvarez R, Olmedo ME, Falagan S, Mielgo X (2020). Clinical-pathological and molecular characterization of long-term survivors with advanced non-small cell lung cancer. Cancer Biol Med.

[6] Malhotra J, Malvezzi M, Negri E, La Vecchia C, Boffetta P (2016). Risk factors for lung cancer worldwide. Eur Respir J.

[7] Wang Y, Ma R, Liu B, Kong J, Lin H, Yu X (2020). SNP rs17079281 decreases lung cancer risk through creating an YY1-binding site to suppress DCBLD1 expression. Oncogene.

[8] Dai J, Lv J, Zhu M, Wang Y, Qin N, Ma H (2019). Identification of risk loci and a polygenic risk score for lung cancer: a large-scale prospective cohort study in Chinese populations. Lancet Respir Med.

[9] Dai J, Shen W, Wen W, Chang J, Wang T, Chen H (2017). Estimation of heritability for nine common cancers using data from genome-wide association studies in Chinese population. Int J Cancer.

[10] Bosse Y, Amos CI (2018). A decade of GWAS results in lung cancer. Cancer Epidemiol Biomarkers Prev.

[11] Sud A, Kinnersley B, Houlston RS (2017). Genome-wide association studies of cancer: current insights and future perspectives. Nat Rev Cancer.

[12] Farh KKH, Marson A, Zhu J, Kleinewietfeld M, Housley WJ, Beik S (2015). Genetic and epigenetic fine mapping of causal autoimmune disease variants. Nature.

[13] Ionita-Laza I, McCallum K, Xu B, Buxbaum JD (2016). A spectral approach integrating functional genomic annotations for coding and noncoding variants. Nat Genet.

[14] Liu B, Montgomery SB (2020). Identifying causal variants and genes using functional genomics in specialized cell types and contexts. Hum Genet.

[15] Qin N, Li Y, Wang C, Zhu M, Dai J, Hong T (2021). Comprehensive functional annotation of susceptibility variants identifies genetic heterogeneity between lung adenocarcinoma and squamous cell carcinoma. Front Med.

[16] Fritsch M, Gunther SD, Schwarzer R, Albert MC, Schorn F, Werthenbach JP (2019). Caspase-8 is the molecular switch for apoptosis, necroptosis and pyroptosis. Nature.

[17] Hashemi M, Aftabi S, Moazeni-Roodi A, Sarani H, Wiechec E, Ghavami S (2020). Association of CASP8 polymorphisms and cancer susceptibility: a meta-analysis. Eur J Pharmacol.

[18] Mandal R, Barron JC, Kostova I, Becker S, Strebhardt K (2020). Caspase-8: the double-edged sword. Biochim Biophys Acta Rev Cancer.

[19] Yuan X, Gajan A, Chu Q, Xiong H, Wu K, Wu GS (2018). Developing TRAIL/TRAIL death receptor-based cancer therapies. Cancer Metastasis Rev.

[20] Wang HB, Li T, Ma DZ, Ji YX, Zhi H (2017). Overexpression of FADD and Caspase-8 inhibits proliferation and promotes apoptosis of human glioblastoma cells. Biomed Pharmacother.

[21] Tummers B, Mari L, Guy CS, Heckmann BL, Rodriguez DA, Ruhl S (2020). Caspase-8-dependent inflammatory responses are controlled by its adaptor, FADD, and necroptosis. Immunity.

[22] Camp NJ, Lin WY, Bigelow A, Burghel GJ, Mosbruger TL, Parry MA (2016). Discordant haplotype sequencing identifies functional variants at the 2q33 breast cancer risk locus. Cancer Res.

[23] Zhao XK, Mao YM, Meng H, Song X, Hu SJ, Lv S (2017). Shared susceptibility loci at 2q33 region for lung and esophageal cancers in high-incidence areas of esophageal cancer in northern China. PLoS One.

[24] Hyland PL, Zhang H, Yang Q, Yang HH, Hu N, Lin SW (2016). Pathway, in silico and tissue-specific expression quantitative analyses of oesophageal squamous cell carcinoma genome-wide association studies data. Int J Epidemiol.

[25] Lin Y, Chahal HS, Wu W, Cho HG, Ransohoff KJ, Dai H (2017). Association between genetic variation within vitamin D receptor-DNA binding sites and risk of basal cell carcinoma. Int J Cancer.

[26] Petrosino M, Novak L, Pasquo A, Chiaraluce R, Turina P, Capriotti E (2021). Analysis and interpretation of the impact of missense variants in cancer. Int J Mol Sci.

[27] Stefl S, Nishi H, Petukh M, Panchenko AR, Alexov E (2013). Molecular mechanisms of disease-causing missense mutations. J Mol Biol.

[28] Capriotti E, Fariselli P, Casadio R (2005). I-Mutant2.0: predicting stability changes upon mutation from the protein sequence or structure. Nucleic Acids Res.

[29] Zhang Z, Wang L, Gao Y, Zhang J, Zhenirovskyy M, Alexov E (2012). Predicting folding free energy changes upon single point mutations. Bioinformatics.

[30] Wang Z, Moult J (2001). SNPs, protein structure, and disease. Hum Mutat.

[31] Yue P, Li Z, Moult J (2005). Loss of protein structure stability as a major causative factor in monogenic disease. J Mol Biol.

[32] Singh SM, Bandi S, Shah DD, Armstrong G, Mallela KM (2014). Missense mutation Lys18Asn in dystrophin that triggers X-linked dilated cardiomyopathy decreases protein stability, increases protein unfolding, and perturbs protein structure, but does not affect protein function. PLoS One.

[33] Liao CY, Yang SF, Wu TJ, Chang H, Huang CF, Liu YF (2021). Novel function of PERP-428 variants impacts lung cancer risk through the differential regulation of PTEN/MDM2/p53-mediated antioxidant activity. Free Radic Biol Med.

[34] Cardano M, Tribioli C, Prosperi E (2020). Targeting Proliferating Cell Nuclear Antigen (PCNA) as an Effective Strategy to Inhibit Tumor Cell Proliferation. Curr Cancer Drug Targets.

[35] Mondal S, Adhikari N, Banerjee S, Amin SA, Jha T (2020). Matrix metalloproteinase-9 (MMP-9) and its inhibitors in cancer: A minireview. Eur J Med Chem.

[36] Chen Q, Huang X, Dong X, Wu J, Teng F, Xu H (2020). Long non-coding RNA ERICH3-AS1 is an unfavorable prognostic factor for gastric cancer. PeerJ.

[37] Jensen K, WuWong DJ, Wong S, Matsuyama M, Matsuyama S (2019). Pharmacological inhibition of Bax-induced cell death: Bax-inhibiting peptides and small compounds inhibiting Bax. Exp Biol Med (Maywood).

[38] Jiang M, Qi L, Li L, Li Y (2020). The caspase-3/GSDME signal pathway as a switch between apoptosis and pyroptosis in cancer. Cell Death Discov.

[39] Ruvolo PP, Deng X, May WS (2001). Phosphorylation of Bcl2 and regulation of apoptosis. Leukemia.

[40] Hengartner MO (2000). The biochemistry of apoptosis. Nature.

[41] Zhao Y, Sui X, Ren H (2010). From procaspase-8 to caspase-8: revisiting structural functions of caspase-8. J Cell Physiol.

[42] von Karstedt S, Montinaro A, Walczak H (2017). Exploring the TRAILs less travelled: TRAIL in cancer biology and therapy. Nat Rev Cancer.

