# Supplementary material for: A causal variant rs3769823 in 2q33.1 involved in apoptosis pathway leading to a decreased risk of non-small cell lung cancer
Source: Cancer Biol Med. 2022 Sep 2;19(9):1385–96. doi: 10.20892/j.issn.2095-3941.2022.0068 (PMC9500222; doi:10.20892/j.issn.2095-3941.2022.0068)
Supplement: Supplementary file 1 [file cbm-19-1385-s001.pdf]

Supplementary materials

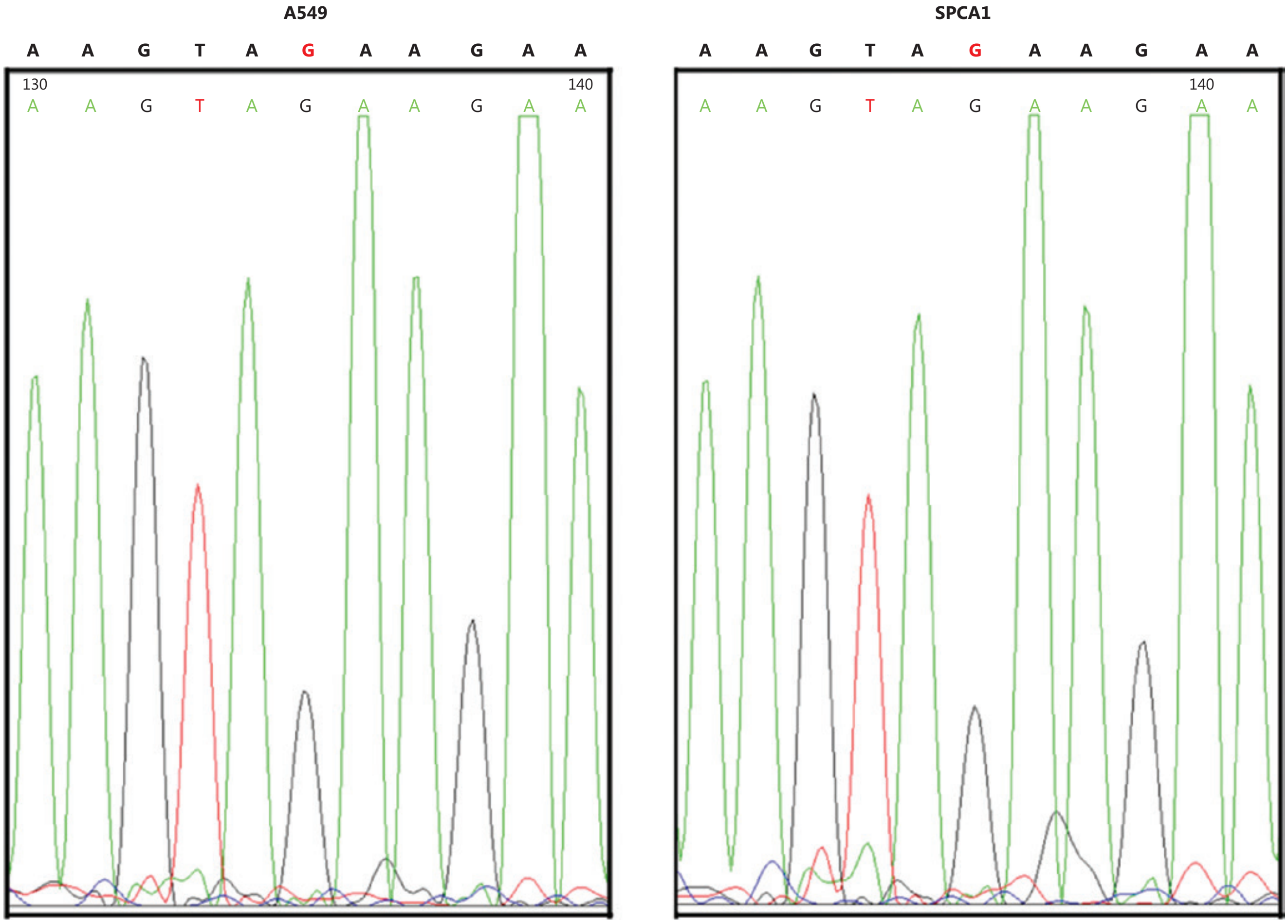

Figure S1 Sanger sequencing of A549 and SPCA1 cell lines.

Table S1 Primers for qRT-PCR

Genes	Forward or reverse	Primer sequence
PCNA	Forward	5′-ATGCCTTCTGGTGAATTTGC-3′
	Reverse	5′-TCACTCCGTCTTTTGCACAG-3′
MMP9	Forward	5′-AAGGCGCAGATGGTGGAT-3′
	Reverse	5′-TCAACTCACTCCGGGAACTC-3′
MMP27	Forward	5′-TTGTTTCTTGTGGCTGCTCA-3′
	Reverse	5′-GCTAAGCCAAAGGAACCCAC-3′
Caspase-3	Forward	5′-CATGGAAGCGAATCAATGGACT-3′
	Reverse	5′-CTGTACCAGACCGAGATGTCA-3′
Bax	Forward	5′-TTCTGACGGCAACTTCAACTGG-3′
	Reverse	5′-CACAGGGCCTTGAGCACC-3′
Bcl2	Forward	5′-CCCGCGACTCCTGATTCATT-3′
	Reverse	5′-CAGTCTACTTCCTCTGTGATGTTGT-3′
β-actin	Forward	5′-CTGGGACGACATGGAGAAAA-3′
	Reverse	5′-AAGGAAGGCTGGAAGAGTGC-3′
